# Risk factors for the presence of dental black plaque

**DOI:** 10.1038/s41598-018-35240-7

**Published:** 2018-11-13

**Authors:** Claudia S. Ortiz-López, Veronica Veses, Jose A. Garcia-Bautista, Maria del Mar Jovani-Sancho

**Affiliations:** 1 Department of Dentistry, Faculty of Health Sciences, Universidad CEU Cardenal Herrera, CEU Universities, Moncada, Spain; 2 Department of Biomedical Sciences, Faculty of Health Sciences, Universidad CEU Cardenal Herrera, CEU Universities, Moncada, Spain; 3 Director of Laboratory Services, Faculty of Health Sciences, Universidad CEU Cardenal Herrera, CEU Universities, Moncada, Spain

## Abstract

In order to evaluate risk factors related to the presence of extrinsic dental black stain, a total of 94 orally healthy volunteers (47 individuals with dental black stain and 47 individuals without dental black stain) were recruited from ten different dental clinics in Valencia and Castellón (Spain). Data regarding their oral hygiene, dietary habits, and oral health status were gathered by questionnaire. Samples of dental plaque, saliva and drinking water were collected for chemical analysis. Three factors were found to be statistically significantly associated with dental black stain, (i) consuming water with high iron content, (ii) consuming water with high pH, and (iii) having a high salivary pH. Other factors such as smoking, taking iron supplements or consuming caffeinated drinks were not found to be risk factors for the presence of black stain. A multivariate logistic regression analysis showed that drinking tap or osmosis-purified water and lower levels of salivary iron increase the risk of having dental black stain. Overall, several risk factors for the presence of dental black stain have been identified. The main modifiable risk factor identified in this study was the consumption of tap or osmosis drinking water.

## Introduction

Extrinsic dental black stain (BS) can appear as a complete or incomplete black line on the buccal and/or lingual surfaces of the teeth near the gingival margin^1^. These stains are firmly attached to the tooth and are difficult to remove with conventional toothbrush and toothpaste. In addition, they tend to reappear after their removal^1^. Published studies show prevalence from 1 to 20%^2,3^. This pigmentation is a relatively common phenomenon in children, where it may occur at any age, even as early as 2 years old^4^.

Although its development is not fully understood, early published literature suggests that BS is a black insoluble ferric compound, formed by the interaction between hydrogen sulfide produced by bacteria and iron^5^. A recent study published by Zhang and collaborators confirms by coupled plasma-mass spectrometry a higher level of iron in BS when compared to standard plaque^6^.

Regarding the bacterial species which could play a role in the formation of BS, initial hypotheses suggested the involvement of chromogenic bacteria, such as *Porphyromonas gingivalis* and other black-pigmented members^7,8^. However, several studies using PCR technology have ruled out the presence of such bacteria^9^, and described a reduced microbial diversity in the black stain as compared to standard plaque, with the enrichment of some bacterial genera (*Actinomyces*, *Cardiobacterium*, *Haemophilus*, *Corynebacterium*, *Tannerella* and *Treponema*) and lower levels of *Campylobacter*, suggesting a dysbiosis as a possible cause or contributing factor to black stain^10^. A recent study published by our group shows the presence of *Tannerella forsythia* in 83,9% of patients with dental BS^11^.

Currently, the main therapeutic approach is frequent dental prophylaxis upon reappearance of the BS, which can cause damage to the enamel microstructure and dental sensitivity. The objective of the present study is to identify risk factors that may influence the appearance of BS in children and adults, such as socio-demographic variables, oral hygiene and status, iron content of saliva and dental plaque, salivary pH, iron levels and pH of drinking water.

## Methods

### Participants

This study was carried out in accordance with the Helsinki guidelines, and approved by the Ethics Committee of CEU Cardenal Herrera University (Authorization Number CEI16/018). The research was carried out in ten private dental clinics in Valencia and Castellón from April 2014 to April 2016. A power analysis based on an expectation a 20% incidence of BS in the population (based on published data)^2^, an alpha value of 0.05 and a power of 0.9 indicated that a total of 90 volunteers was required^12^. To account for losses during to follow up, 47 patients with black plaque of bacterial origin in at least 2 teeth (according to the diagnostic criteria proposed by Koch *et al*.^13^) were recruited to participate in the study. Equal numbers of age- and sex-matched subjects without BS were selected as control group, making a total of 94 participants. All adults and legal guardians of participant children gave informed consent. The only exclusion criterion was to have taken antibiotics 15 days prior to the dental appointment.

### Questionnaire

Each participant completed a questionnaire, including questions regarding socio-demographic variables such as age and sex, hygiene habits such as the type of dental brush or numbers of brushes per day, dietary habits such as snacking between meals, iron supplementation, and type of drinking water consumed. Other chemical parameters such as pH of saliva and drinking water and iron content in plaque, saliva and drinking water were also recorded.

### Clinical examination

Intraoral examination of each patient was performed to evaluate the presence of black stain and to determine the degree of pigmentation, according to the classification scheme described by Koch^13^ and modified by Gasparetto *et al*.^14^, as reported previously by our lab^11^. Each patient’s BS score was assigned as follows: 1 for pigmented spots or incomplete lines parallel to the gingival margin; 2 for solid lines pigmented, readily observable and limited to the cervical third of the tooth surface; and 3 for pigmentation extending beyond the cervical third. The evaluation was always performed by the same dentist. The values obtained were analyzed by a test-retest study to assess intra-operator agreement. Briefly, ten images of participants with black staining were randomly selected and the teeth were measured twice (with an interval of ten days) by the same operator. The Kappa index obtained was 0.788, indicating good reliability of the method. Oral health status of each participant was assessed by calculating the DMFT (decay-missing-filled-teeth) Index, Periodontal Index and Hemorrhage Index^15^.

### Analysis of dental plaque, saliva and drinking water samples

**Figure 1:**
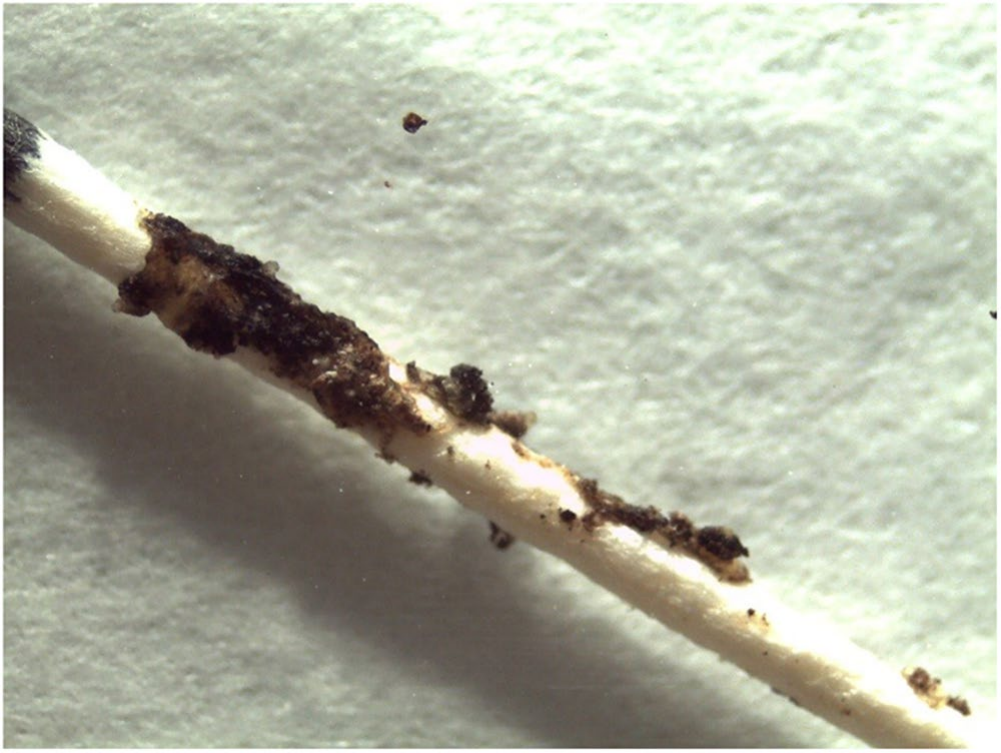
Microscopic image of dental black stain collected with an endodontic paper point.

Supragingival dental plaque samples were collected by scraping with a plastic scaler from tooth surfaces taking care to avoid unnecessary removal of enamel^16^. A total of 1 mg of each collected plaque sample was transferred to sterile endodontic paper points and stored in sterile 0.5 ml DNA- and RNA-free Eppendorf tubes, and store at −20 °C until processed, as described previously^11^ (Fig. 1). Unstimulated whole saliva samples were collected as described elsewhere^17^. Each participant provided a sample of drinking water (for those drinking tap water) or specified the brand of mineral water consumed. Supragingival dental plaque samples were subject to digestion with 65% nitric acid (JT Baker®, Fisher Scientific, Madrid, Spain) for 24 hours prior to iron determination^18^. Iron determination in all samples (saliva, drinking water and digested supragingival plaque) was performed using Spin 200E equipment (Spinreact®, Girona, Spain), following the manufacturer instructions, and as described elsewhere^19^. Salivary and drinking water pH was analyzed with a pH-meter Basic 20 with glass electrode for microsamples (Crison instruments®, Barcelona, Spain).

### Statistical analysis

Pearson’s chi-squared test was used to analyze the distribution of black stain amongst participants and the assessment of risk factors such as hygiene and dietary habits, smoking or type of drinking water. The Mann-Whitney test was used to test for differences between the BS and control groups with regard to the oral health status (DMFT index, bleeding index, periodontal index) and chemical variables (salivary and drinking water pH, iron levels in saliva, dental plaque and drinking water). Spearman’s coefficient was used to analyze correlations. Logistic regression was used to perform a multivariate analysis of the different risk factors assessed.

### Ethical approval

All procedures performed in studies involving human participants were in accordance with the ethical standards of the institutional and national research committee and with the 1964 Helsinki Declaration and its later amendments or comparable ethical standards. The study was approved by the Ethics Committee of CEU Cardenal Herrera University (Authorization Number CEI16/018).

### Informed consent

Prior informed consent was obtained from all individual participants included in the study.

## Results

A total of 94 volunteers participated in the study. The mean age of the control group was 39.8 ± 18.1 years, and 39.9 ± 18.3 years for volunteers with BS (Table 1). Age showed no association with the presence of black stain (*P* = 0.982). The ratio of female to male volunteers was calculated as 1.69: 1. No statistically significant differences was found between males and females with BS, ruling out sex as a risk factor (*P* = 0.831; Table 1). The mean number of stained teeth for participants with BS was 17.68 ± 6.47, with 25 surfaces ± 11.01 stained on average, with a higher number of lingual surfaces stained (63.9% of lingual surfaces were stained surfaces whilst 35.2% of vestibular surfaces showed presence of BS; data not shown). Age was found to be inversely related to the percentage of stained surfaces. As age increases, the percentage of stained surfaces decreases, with a Spearman’s rho of −0.381 (*P* = 0.029).

**Table 1:** Demographic characteristics of the population of study volunteers, including the sex distribution and mean age (SD).

	Control	Black Stain
Male % (n)	38.3 (18.0)	36.2 (17.0)
Female % (n)	61.7 (29.0)	63.8 (30.0)
Mean age in years (SD)	39.8 (18.1)	39.9 (18.3)

### Risk factors related to oral hygiene, dietary and social habits

Participants were asked about their oral hygiene habits, such as frequency of tooth brushing and type of dental brush employed. No significant differences were found amongst participants brushing three or more times when compared to participants brushing twice or once per day (*P* = 0.321). Similarly, the type of dental brush used (manual or rotatory, or combination of both) did not show an impact upon the presence of BS (*P* = 0.553; Table 2). Participants were asked about several dietary habits. The BS group showed a significantly lower number of participants who snacked between meals when compared to the control group (31.9% vs 53.2%, *P* = 0.037; Table 2). The majority of participants in the control group consumed mineral water (93.6%) whilst in the BS group 57.4% of participants consumed mineral water, 27.7% consumed tap water, and 14.9% consumed osmosis-purified water (Table 2). Only 6.4% of participants in the control group drank osmosis-purified water and none of them referred to drinking tap water. When asked about the consumption of iron supplements, 8.5% and 6.4% of control and BS participants, respectively, indicated that they consumed regularly iron supplements (Table 2). Pearson’s chi-squared test was applied to the above dietary factors, resulting in a *P* value of 0.037 for snacking between meals (being these a protective factor) and a *P* < 0.001 for the type of drinking water consumed (with consumption of tap or osmosis water being a risk factor for the presence of BS).

**Table 2:** Assessment of risk factors related to oral hygiene, diet and social habits of the participants.

	Control (% of participants)	Black Stain (% of participants)	*P* value
Brushing Technique			0.553
Manual	63.8	66.0	
Manual/rotatory	12.8	6.4	
Rotatory	23.4	27.7	
Number of brushes per day			0.321
1	21.3	34.0	
2	42.6	40.4	
3 or more	36.2	25.5	
Snacking between meals			
Yes	53.2	31.9	**0.037**
Source of drinking water			**<0.001**
Mineral water	93.6	57.4	
Tap water	0	27.7	
Osmosis water	6.4	14.9	
Iron supplements			0.500
Yes	8.5	6.4	
Smoking			0.247
Yes	19.1	10.6	
Caffeine consumption			0.294
Yes	85.1	76.6	

Regarding social habits, 19.1% of control participants were smokers, whilst 85.1% of control participants consumed regularly caffeinated drinks (tea, coffee and/or soft drinks). In the BS group 10.6% of participants consumed tobacco, whilst 76.6% of participants drank caffeinated drinks habitually. No statistically significant differences were found regarding the consumption of iron supplements, smoking or caffeine consumption (*P* = 0.500; *P* = 0.247; *P* = 0.294, respectively).

### Risk factors related to oral health status

Oral health status of the participants was assessed with the DMFT index and the bleeding and periodontal indexes. The control group showed a median DMFT index of 7 whilst the BS group showed a median DMFT value of 5 (Interquartile range 9 and 8 respectively; Table 3). BS patients showed an increased bleeding index (median of 4.3 in the BS group versus 3.4 in the control group). The periodontal index was obtained for all adult participants, with similar results in both groups (median 16.8 in both groups, with interquartile ranges of 1.20 and 1.00 (control and patients respectively).

**Table 3:** Assessment of risk factors related to the oral health status of the participants, including DMFT, bleeding, and periodontal (only adult participants) indexes (Medians; interquartile range).

Oral Health Status	Control	Black stain	*P* value
DMFT index	5; 9	7; 8	0.298
Bleeding index	4.3; 5.5	3.4; 6.1	0.574
Periodontal index	16.8; 1.2	16.8; 1.0	0.416

Mann-Whitney test was applied, since the above mention parameters showed non-normal distribution. No statistically significant results were found when comparing the control and the BS groups, with *P* values of 0.298, 0.416 and 0.574 respectively for DMFT, periodontal and bleeding indexes (Table 3).

### Risk factors associated with salivary iron and pH, the iron in dental plaque and the pH and iron levels of drinking water

Salivary pH was measured in all study participants, with an average value of 7.2 ± 0.7 in the control group versus an average value of 8.0 ± 0.5 in the participants with BS (Table 4). The Mann-Whitney test demonstrated a highly significant difference in these salivary pH values (*P* < 0.001). Iron levels were also quantified in saliva of the volunteers; control participants carried an average of 63.9 ± 93 µg/dl, whilst the BS participants showed lower levels of salivary iron, with an average value of 5.4 ± 11.1 µg/dl. A similar trend was detected in the iron levels in dental plaque, BS participants showed a lower level of iron (77.0 ± 67.5 µg/dl) than control participants (116.4 ± 91.4 µg/dl). Both iron levels in saliva and dental plaque were found not to be statistically significant (*P* = 0.066; *P* = 0.153; Mann-Whitney test).

**Table 4:** Assessment of risk factors related to the pH and iron levels in saliva, dental plaque and drinking water.

	Control	Black stain	*P* value
Salivary pH	7.2 ± 0.7	8.0 ± 0.5	<**0.001**
Salivary iron	63.9 ± 93.0 µg/dl	5.4 ± 11.1 µg/dl	0.066
Iron in dental plaque	116.4 ± 91.4 µg/dl	77.0 ± 67.5 µg/dl	0.153
Drinking water pH	6.9 ± 1.0	7.4 ± 1.4	**0.003**
Iron in drinking water	20.7 ± 51.7 µg/dl	70.8 ± 77.6 µg/dl	**0.028**

Levels of pH and iron were analyzed in the drinking water consumed by the participants. We found that pH was lower in the control group (6.9 ± 1.0) when compared to the average pH of drinking water in the BS group (7.4 ± 1.4; *P* = 0.003). Iron levels were also lower in the drinking water consumed by the control participants (20.7 ± 51.7 µg/dl control group vs 70.8 ± 77.6 µg/dl BS group, *P* = 0.028).

### Multivariate analysis

Multivariate logistic regression analysis revealed that BS was statistically significantly associated with the type of drinking water (tap/osmosis) and the presence of iron in saliva (lower quantity; Table 5). The odds of having BS was 13 times higher in participants consuming tap or osmosis water, and salivary iron consistently influenced the tendency for BS with an odds ratio of about 2.25% for each unitary increase in iron levels (Table 5).

**Table 5:** Multivariate analysis: model of risk factors for the occurrence of BS. Dependent variable: presence of BS in at least two teeth.

Independent variable	Estimated OR	Significance level	95% CI
Salivary iron	0.975	0.018	0.955–0.996
Tap/osmosis drinking water	13.126	0.001	2.948–58.441

## Discussion

This study aimed to identify significant risk factors for the presence of dental black stain. According to previous published studies, several factors were investigated such as demographic characteristics^20^, oral hygiene and oral health status^3,21^, dietary factors such as snacking between meals and type of drinking water^3,22^, intake of iron supplements and iron levels of saliva and dental plaque^6^, and pH and iron contents of saliva^23,24^. Our study included adult participants; hence factors such as smoking or caffeine consumption were also included as potential risk factors for the presence of black stain in adults.

We found no association between sex and the presence of BS, in agreement with the majority of published work^13,20,22,25^. Similarly, we did not find association between age and the presence of BS, coinciding with previous reports^13,22,25^. One study by Chen and coworkers showed a correlation (although not statistically significant) between age and BS^20^, reporting also that the number of stained teeth increased with age, contradicting our findings. This could be due to the different population analyzed, since the mean age of the participants in the Chen study was 4.55 years and our study included adult participants with a mean age of 39.85 years.

Several studies have indicated in past years that BS could be associated with lower caries^3^. Our results also found that DMTF indexes were slightly lower in BS participants, although the difference was not found to be statistically significant. Early studies of BS suggested a role for chromogenic bacteria as possible causative factor of this extrinsic tooth discoloration^7,8^. Since some of these bacteria also related to periodontal disease, it has been suggested that gingival bleeding stimulated by the presence of the bacteria could be the source of iron necessary for the formation of the black pigment^26^. Even though bleeding indexes were elevated in the BS group, no substantial differences amongst participant groups were detected, ruling out periodontal bleeding as a risk factor for BS, in agreement with recent literature attributing better oral health to individuals with BS^3,8,20,22^. Some authors have pointed out that individuals with BS might demonstrate better oral hygiene, in an attempt to control BS extension, which in turn will lead to improved oral health. Although there is conflicting evidence about the influence of oral hygiene in BS, oral hygiene habits were not found significantly different amongst participant groups in our study.

When dietary and social habits were investigated, we found no association between habits such as smoking, intake of caffeinated drinks or iron supplements. A recent report by Prskalo and coworkers highlights the consumption of beets as risk factor for BS^27^ whilst intake of collard greens and wine were more frequent in participants without BS. Prskalo’s results also showed no association between intake of iron supplements and BS^27^. Our control group showed higher frequency of snacking between meals when compared to the BS group. One dietary factor was identified as a strong risk factor, the type of drinking water. In agreement with one previous study published in Brazil^22^, we found that in our study population, consuming tap or osmosis water increased the odds of having BS by 13 times.

Levels of pH and iron concentration in saliva have been reported to differ between the subjects with and without caries, with a significant negative relationship between the salivary pH and iron concentration^28^. Lower salivary pH and higher iron levels have been postulated as increasing caries risk^28^. Since BS is traditionally associated with reduced caries formation, we decided to investigate pH levels and iron concentration in saliva and dental plaque, to find out if any of the above factors could play a role in promoting BS formation and maintenance. Our BS participants showed increased salivary pH and lower salivary and plaque iron levels, as reported previously^23,24^. Snacking decreases salivary pH regularly, so this could explain our finding that snacking between meals could play a protective role against BS, due to the effect on salivary pH, promoting acidification.

Both pH and iron levels were analyzed in drinking water, aiming to identify if different levels of pH or iron could be the underlying reason for the increased BS risk detected in participants consuming this type of water. We found higher pH levels, but also, higher iron content in tap/osmosis water than in mineral water. One explanation for this discrepancy between salivary and drinking water iron levels could be that lower pH could enhance iron solubility and higher pH will have the opposite effect^29^. Even though tap/osmosis water provides a higher iron content, elevated salivary pH would reduce the availability of this iron to bacteria in the gingival sulcus, hence modifying the normal microbiota, leading to the dysbiosis observed in BS^10^.

The main limitations of our study are related to the in-depth analysis of dietary factors involved in BS. More cohort studies following participants with substantially different diets would be necessary to address this lack of information with respect to the role of diet in BS. Major strengths of our study are the inclusion of adult participants (only one previous study included volunteers up to 50 years old)^30^ and the identification of one preventable risk factor for the presence of black stain; the consumption of tap/osmosis water.

Three factors were found to independently influence the presence of BS (consuming water with high iron content, consuming water with high pH, and having a high salivary pH). The two main factors associated with the presence of BS were low levels of salivary iron and consuming tap/osmosis water. Patients with BS should be advised to switch to mineral water to reduce the risk of BS reappearance following dental prophylaxis.
